# Medical Oncology

**DOI:** 10.1080/13577140120097102

**Published:** 2001-11


### MEDICAL ONCOLOGY

### 010 The SSG Register – Tool for Clinical Research

H C Bauer[1], C S Trovik[2], T A Alvegård[3], P Gustafson[4], B Skytting[5], A Rydholm[4]

*[1]Oncology Service Dept. of Orthopedics Karolinska Hospital, [2]Dept. of Orthopedics Haukeland University Hospital , [3]Dept. of Oncology Lund University Hospital , [4]Dept of Orthopedics Lund University Hospital, [5]Dept of Orthopedics Soder Hospital*

*Objective:* Present how the SSG register of bone and soft tissue sarcoma patients has an important role for ensuring quality control and as a basis for research projects.

*Methods:* The SSG Register was initiated in 1986 and more than 5000 patients from sarcoma centers in Finland, Norway and Sweden have been accrued. Patient data was registered at the participating institutions. For in-depth studies, clinical data was reviewed and completed and the histopathology reclassified by the SSG Pathology Board.

*Results:* For quality control, changes in referral and treatment of soft tissue sarcoma was assessed in 1851 soft tissue sarcoma patients treated 1986–97. 63% were referred before open biopsy or excision. Referral improved for deep-seated lesions but not subcutaneous. Fine-needle aspiration for cytologic diagnosis was used in 81%. The amputation rate decreased from 15% to 9%, surgical margins did not improve, but the overall use of radiotherapy increased from 20% to 30%. Studies of local recurrence of soft tissue sarcoma was based on 1613 patients treated 1986–95. The local recurrence rate was 17%, but for high-grade deep-seated lesions 26%. After a first local recurrence, the risk of subsequent recurrences was 30%, and the overall risk of amputation for local recurrence was 22%. Local recurrence was associated with a high risk of subsequent metastases but did not appear to be an important cause of metastases. Studies on synovial sarcoma was based on 104 patients 1986–94. In a population based study of all patients in Sweden during this time-period, the median age was 38 years, and adjusted for age, the highest incidence was in 50–60 year olds. The 5 and 7 years survival rates were 0.76 and 0.69. Large size, local recurrence, Grade IV, MIB1 index > 10, and SYT-SSX fusion transcript was associated with metastases.

*Conclusion:* These studies show that a multi-institutional sarcoma registry can be used both for monitoring treatment and as a basis for in-depth studies. Based on these studies, guidelines for radiotherapy in soft tissue sarcoma have been changed and we have been able to show that referral to sarcoma centers have improved, leading to lower local recurrence rates.

### 014 RTOG 95-14: A Phase II Study of Neoadjuvant Chemotherapy (CT) and Radiation therapy (RT) in the Management of High Risk, High Grade, Soft Tissue Sarcomas (STS) of the Extremities and Body Wall: A Preliminary Report

W G Kraybill[1], I J Spiro[1], J Harris[1], D S Ettinger[1], A Trotti[1], D R Lucas[1], R Blum[2], B R Eisenberg[1]

*[1]Radiation Therapy Oncology Group, [2]Eastern Cooperative Oncology Group*

*Objective:* Introduction and Objectives: Previous reports have demonstrated improved disease- free survival in patients with large high risk STS given neoadjuvant chemotherapy, radiation therapy and post resection chemotherapy when compared with matched controls not given chemotherapy. To evaluate this in the multi-institutional setting, the Radiation Therapy Oncology Group (RTOG) instituted a Intergroup Phase II trial of multi-agent CT and preoperative RT combined with resection in patients with high-grade, high-risk STS of the extremities and body wall. Other objectives were to develop a sarcoma working group for future Phase II and Phase III clinical trials and to develop a tissue repository for future studies.

*Methods:* Methods: To assess toxicity, response rates, local control, distant recurrence, survival and complications, 66 patients with primary high grade (II or III of III) STS > 8 cm in maximum diameter were treated with high-dose neoadjuvant CT (Modified MAID) plus G-CSF and preoperative RT and 3 cycles of postoperative CT plus G-CSF. RT 44Gy was given in split courses of 22GY between cycles of CT.

*Results:* Results: The first 45 patients had potentially 1-year follow-up and were eligible for analysis. Of which, forty-two MO patients were analyzed. Median tumor diameter was 13 cm (Range 8–55). Thirty-four (81%) were Grade 3 tumors and 8 (19%) were Grade 2 on a 3 grade system. Median follow-up was 2.35 years (0.23–3.53). Thirty-five patients had clear surgical margins. Seven patients had persistent disease with 4 having involved margins at the time of resection, 1 patient having unresectable tumor due to progression, and 2 patients requiring amputation secondary to disease. Two patients have relapsed locally, 3 patients have relapsed in the lungs, 1 patient developed metastases in the right shoulder and 2 patients developed a second primary. Six patients have died, 3 due to recurrent sarcoma, 2 due to a second primary, and 1 for reasons pending clarification. The observed toxicities were 76% (32) Grade 4 hematologic toxicity and 24% (10) Grade 4 non-Hematologic toxicity. Twenty-nine patients (69%) experienced Grade 4 neutropenia and 12 (29%) Grade 4 thrombocytopenia. Grade 4 skin toxicity in 5 patients (12%) was the most common non-hematologic toxicity. While these expected toxicities are significant, 85% of patients completed preoperative CT, 93% of patients completed preoperative RT and 75% completed postoperative chemotherapy. Delayed wound healing was reported in only 10 patients (26%). Estimated 2-year survival in these high-risk tumors was 95%. There were 42 patients in the survival analysis and only 40 patients in other analysis. There is only survival data on 2 patients. Of the 21 patients excluded from the analysis because they were entered after April 1999, 16 (76%) have experienced grade IV toxicity. Three of 21 (14%) have died, all of causes related to the cancer under study.

*Conclusions:* While this is a preliminary report, the estimated 2-year survival in these very high-risk patients is encouraging with tolerable toxicity.

### 025 Successful Treatment of High Grade Soft Tissue Sarcoma with Induction (Neoadjuvant) Chemotherapy: Clinicopathological Analysis of Thick Capsule Formation Allowing Less Extensive Surgical Resection

Felasfa Wodajo, James C Wittig, Dhruv Kumar, Dennis Priebat, Robert M Henshaw, Martin M Malawer

*Washington Cancer Institute Washington Hospital Center*

*Objective:* The histologic changes occurring in the periphery, or pseudocapsule, of sarcomas after induction chemotherapy have not previously been characterized in detail. We have used neoadjuvant chemotherapy in lieu of preoperative radiation therapy for limb sparing resections of large, high grade soft tissue sarcomas. Microscopic examination of soft tissue tumors that have responded well to induction chemotherapy demonstrates a thick, well defined "capsule" surrounding necrotic tumor.

*Methods:* During the period of 1988 to the present, over 60 resections for high-grade soft tissue sarcoma were performed by the same surgeon. Nearly half were eligible for enrollment in an induction chemotherapy protocol consisting of intravenous adriamycin,

DOI: 10.1080/13577140120097102

ifosfamide and intra-arterial cis-platinum. Resection specimens from ten patients were selected for histological analysis. Six of these were high grade tumors with median tumor necrosis of 94%. Two were high grade, poor responders (avg. 40% necrosis) and two were high grade not having undergone neoadjuvant treatment. Tumor types included MFH (3), liposarcoma (2), leiomyosarcoma (2), MPNST (1) and fibrosarcoma (1).
*Results:* In 5/6 patients with good therapeutic response, the tumor pseudocapsule was converted into an outer zone of densely collagenized fibrous tissue resembling a true “capsule” and an inner zone of loose vascularized fibrous tissue. The outer zone did not contain viable tumor cells. Untreated patients and poor responders demonstrated thin, interrupted and poorly defined outer zones and similarly vascular inner zones.
*Conclusion:* Limb sparing resection for soft tissue sarcoma in the extremities often results in close surgical margins near critical neurovascular structures. Preoperative chemotherapy resulting in formation of a thick, collagenized capsule facilitates resection by forming a safe biological border for dissection. This treatment strategy may be considered analogous to induction chemotherapy in osteosarcoma, which has permitted an increased rate of limb salvage without increasing the rate of local recurrence.

**041 A Retrospective Review of Mayo Clinic's Phase II Clinical Trials of Advanced Sarcomas to Examine Survival Differences between Leiomyosarcomas of the GI Origin (GIST) Versus Non-GI Origin**

S H Okuno, M R Mahoney, A M Olivera
*Mayo Clinic*

*Objective:* Recent reports suggest that patients with metastatic GIST have inferior survival compared to patients with non-GI leiomyosarcoma. Previously GISTs were classified as leiomyosarcomas of the GI tract and clinical experience has shown that these tumors of the gut behaved differently to treatment regimens than similar tumors of non-GI origin. GISTs are now classified as mesenchymal tumors of Cajal cells which express c-kit and are responsive to the small molecule STI-571. We wanted to verify if survival differences were evident in our leiomyosarcoma patients (GIST vs. non-GIST).
*Methods:* We reviewed from our previous 12 phase II studies for advanced sarcoma and identified 93 of 430 patients diagnosed with leiomyosarcoma. These patients were entered into the phase II studies from 1971-1992 and largely when surgical options were exhausted. Treatments included adriamycin; methyl-CCNU, actinomycin D, cytoxan, vincristine; adriamycin, imidazole carboxamide, vincristine; pyrazofurin; cytoxan, adriamycin, cisplatin; maytansine; mitomycin, adriamycin, cisplatin; menogaril; interferon gamma; ifosfamide, etoposide; ifosfamide, mitomycin, adriamycin, cisplatin; and taxotere. Because of limited samples and age of the tissue, staining for c-kit (CD 117) was not performed.
*Results:* Thirty-six (39%) cases were classified as GIST (vs. 57 non-GIST). Ninety-one patients have died. Kaplan-Meier estimates are as follow:

| Rate | GIST-Estimate (95%) | Non-GIST-Estimate (95%) |
|---|---|---|
| Median | 1 yr (0.5-1.4 yrs) | 1 yr (0.5-1.6 yrs) |
| 1-year | 53% (39-72%) | 51%(39-66%) |
| 2-year | 11%(4-28%) | 32%(22-46%) |
| Log-rank, p-value=0.45 | | |

*Conclusion:* In our retrospective review of patients on 12 advanced sarcoma Phase II clinical trials, we observed no statistically significant difference in survival between patients with advanced leiomyosarcomas of the GI versus those of non-GI origin.

**045 Sequential High-Dose Doxorubicin (DX) and High-Dose Ifosfamide (IF) in Advanced Previously Untreated Soft Tissue Sarcomas (STS). A Multicenter Phase II Study of the Spanish Group for Research on Sarcomas (GEIS)**

Joan Maurel, Antonio Lopez-Pousa, Jose Maria Buesa, Xavier Garcia del Muro, Carme Bala a, Antonio Casado, Javier Martinez-Trufero, Ramon De las Pe as, Javier Martin, Joaquin Bellmunt
*Hospital Clinic Barcelona*

*Objective:* DX and IF the most active drugs in adult STS have a dose-response effect limited by its hematological toxicity when used in combination. We have designed a first-line sequential trial of high-dose DX followed by high dose IF to maximize dose-intensity and reduce toxicity.
*Methods:* Pts with advanced STS without prior exposure to chemotherapy were included. Prior radiotherapy was allowed if given to non-indicator lesions. Gastrointestinal sarcomas (GIST) and pts over 65 years were excluded. Treatment consisted of DX 90 mg/m$^2$ q 14 days for 3 cycles followed by IF at 12.5 gr/m$^2$ q 21 days for 3 cycles, both regimens with G-CSF support.
*Results:* From Dec’98 to May’01 we enrolled 56 STS pts, 53 were eligible (8 locally unresectable, 45 metastatic). Median age was 52 years (range 24-65). performance status (PS) was 0 in 20 pts, 1 in 29 and 2 in 4. Metastatic sites: lung 31 pts (only lung 11 pts), nodes 9, peritoneal 8, liver 7, bone 6. No. of sites: 1 in 32 pts, 2 or more in 13. 43 patients are already evaluate for toxicity and 41 for response (2 died without radiological assessment). Median DX dose was 43 mg/m2/w, 29 pts (68%) full dose, with grade III/IV toxicity: neutropenia 9 pts (21%), neutropenic fever 3 pts (7%), mucositis 12 pts (28%).There were 13 (32%) partial responses (PR). 8 pts did not receive HDI (3 progressive disease and poor PS, 3 patients with severe no treatment-related infection and 2 refused). Dose-intensity HDI was 3.1 gr/m$^2$/w, with grade III/IV neutropenia 15 pts (42%), neutropenic fever 8 pts (23%) and asthenia 10 pts (28%). After a full course of therapy 20 pts (48%) achieved a PR. Two pts with a PR to DX progressed under HDI, and 9 no responders to DX (8 SD and 1 PD) obtained a PR with HDI. There were no toxic death.
*Conclusion:* This sequential dose-dense schedule is feasible, has an acceptable toxicity profile and has a response rate similar to concomitant high dose IF-DX regimens.

**049 Association between Breast Cancer and Cartilaginous Tumors: Phenotypic Characterization of a Hitherto Unrecognized Potential Hereditary Trait**

M. Timmerman[1], A. M. Cleton-Jansen[1], M. J. v.d. Vijver[1], C. van Asperen[2], L. C. v.d. Broek[1], P. C. Hogendoorn[1]
[1]*Department of Pathology, Leiden University Medical Center,*
[2]*Department of Medical Genetics, Leiden University Medical Center*

*Objective:* Recently we documented a strong association between the occurrence of cartilaginous tumors (enchondroma, chondrosarcoma) and breast cancer in the same patient, using a nation-wide case-control study. This study revealed an odds ratio of 7.62 for a potential association of breast and cartilaginous tumors, pointing statistically strongly towards a genetic trait. This is furthermore corroborated by the age of onset in patients with breast cancer as the first tumor, which is about 10 years earlier than breast cancer in the general population. Following this statistical/epidemiological analysis we report on the phenotypic characterization of the patient group.
*Methods:* The Dutch BRCA1 and BRCA2 family database was searched for cases recording a cartilaginous tumor. Moreover using the national pathology database the tissue blocks of all patients reported to fulfil the associated tumors mentioned were retrieved. Reported diagnoses were reviewed; breast cancer specimens were classified and histologically characterized according to the procedures used by the Breast Cancer Linkage Consortium. In addition the cartilaginous tumors were analyzed with emphasis on the central versus peripheral localization in the skeleton as previous studies proved a different molecular mechanism to be operative in the different subtypes.
*Results:* In the Dutch BRCA1 and BRCA2 database no case of chondrosarcoma nor enchondroma was reported, neither within the same patient nor within the pedigree pointing to a trait which is different from the aforementioned breast cancer syndromes. Remarkably all cartilaginous tumors are of one common histological subtype being centrally localized whereas no peripheral cartilaginous tumor was registered. The breast tumors were histologically heterogeneous with varying differentiation grade. Results on immunohistochemical staining (p53, Bcl2, Her2-neu, p16, p21 estrogen and progesterone receptor and E-cadherin) will be presented.
*Conclusion:* Evidence is presented that the recently described association between the occurrence of breast cancer and cartilaginous tumors is different from other known breast cancer syndromes with a restricted spectrum of cartilaginous tumors.

**057 Temozolomide as a 6-Week Continuous Oral Schedule in Advanced Soft Tissue Sarcoma: A Phase II Trial of the Spanish Group for Research on Sarcomas (GEIS)**

X Garcia del Muro, A Lopez Pousa, J M Buesa, J Martin, A Poveda, I Bover, P Escudero, J Martinez Trufero, A Casado
*Institut Catala d'Oncologia*

*Objective:* Temozolomide is an oral imidazotetrazine derivative, that lacks activity against soft tissue sarcoma (STS) at standard doses. Extended continuous oral administration mimics continuous infusion, and increases drug exposure (*Ca Res* 1998;58:4363).
*Methods:* A multicenter phase II study was performed to assess the activity and toxicity of Temozolomide administered at a dose of 75 mg/m$^2$/day, as a 6-week oral continuous schedule every 9 weeks, in adult patients (pts) with previously treated STS. From November 1999 to date, 30 pts with advanced STS and non-irradiated measurable lesions were included into the study.
*Results:* At present, 27 pts were evaluable: 2 were inelegible and 1 refused treatment. Median age was 51 (29–76) years; 11 male and 16 female; PS: 0:9, 1:14, 2:4 pts. Histologic types were: GIST:4, MFH:5, fibrosarcoma:3, leiomyosarcoma:4, liposarcoma:2, angiosarcoma:2, mixed mullerian tumor:2, and others:5. Grade: III:15, II:9, I:3 pts. Prior regimens included doxorubicin and Ifosfamide in 26 and 25 pts, respectively. A total of 42 cycles were administered. Six pts did not receive a complete cycle of treatment due to: 3 rapid progression, 2 early death from disease, 1 pulmonary thromboembolism, and they were considered as treatment failures. There were 4 PR and 3 SD (Overall response rate: 15%, 95% CI: 4–34). Partial responses were seen in 2 pts with uterine leiomyosarcoma, 1 mixed mullerian tumor and 1 GIST, and they lasted 14, 10+, 7 and 4 months. Hematological toxicity was: Neutropenia G-IV:1, G-III:1, G-II:2 pts, Thrombocytopenia GIII:2, GII:3 pts, Anemia G-III:4, G-II:3 pts). Non-hematological toxicity was: Nausea G-II:3 pts, Vomiting G-II:4 pts, Asthenia G II:4 pts.
*Conclusion:* Temozolomide at this extended continuous schedule has activity against STS, appears to be well tolerated, and deserves further evaluation, specially against uterine sarcomas.

**059 An Overview of Treatments Results in Osteosarcoma: Is there a need for a Global Collaboration?**

Sigbjørn Smeland
*Norwegian Radium Hospital*

*Objective:* To overview the treatments results in high-grade osteosarcoma and to outline the need for international collaboration to obtain further improvements.
*Methods:* Review the literature of treatment results in high-grade osteosarcoma. Published results from prospective phase II studies including more than 100 patients and randomized studies are included in this analyses.
*Results:* High-grade osteosarcoma are rare tumors that make up approx. 0.1 % of all malignant tumors. Introduction of intensive chemotherapy has significantly improved the prognosis for patients with non-metastatic disease and most patients with extremity localized tumors are currently operated with limb saving procedures. However, there have been no major improvements in outcome for the last 15 years and subgroups of patients still harbor a very poor prognosis. Most progress in osteosarcoma treatment is obtained from prospective phase II studies in patients with non-metastatic extremity localized tumors in patients aged < 40 years (classical osteosarcoma). The best centers report a 5-year overall survival of 70% and currently more than 80% of the patients are operated with a limb saving approach. Poor prognostic factors at time of diagnosis include presence of metastatic disease, axial localization and large tumor volume. Age is not a consistent prognostic factor in osteosarcoma. Surgical margins, tumor necrosis and treatment duration are important treatment related factors. Due to difference in post-operative therapy the true impact of tumor necrosis is hard to assess; in large randomized trials with similar post-operative therapy to good and poor responders the differences in event-free survival are in the range from 25 to 40%. Most centers agree that a three-drug combination with methotrexate, doxorubicin and cisplatinum should be given up-front to all patients and several groups also include ifosfamide to all or subgroups of patients in primary treatment. The schedule of therapy is still being investigated and important issues yet to be answered are the optimal use ifosfamide and cumulative dose of doxorubicin. Such answers are likely only to be obtained in randomized trials. The lack of progress in treatment of osteosarcoma for the last 15 years justify the introduction of novel treatment approaches and specially to patients with an inherently poor prognosis. Candidate agents include interferon-a and the immunostimulator, muramyltripeptide. For relapsed patients the prognostic factors important for outcome are in addition to surgical remission, extent of and time to relapse and application of adequate second-line chemotherapy. The benefit of radiotherapy for patients with inoperable disease/intralesional surgery is debated and needs further elucidation. Even with maximal doses of chemotherapy, the prognosis for patients with primary metastatic disease is dismal and there is no support in current literature for the use of myeloablative chemotherapy. In the Scandinavian Sarcoma Groups database containing 485 patients with osteosarcoma, 30% of the patients belongs to a heterogeneous group of patients with non-extremity localized tumors and/or secondary osteosarcomas and/or age> 40 years, that up to date have been excluded from most (inter)national trials. The treatment principles are based on data obtained from trial conducted on mainly classical osteosarcomas assuming a similar biology for the whole group of patients.
*Conclusion:* The rarity of osteosarcoma strongly argue for international collaboration to obtain further progress in treatment and insight in the biology of the disease. In primary treatment randomized trails are needed both to optimize the scheduling of the currently known active drugs and to test out novel treatment agents. For non-classical osteosarcomas and relapsed patients international prospective phase II studies are needed to test out treatment regimens and to identify prognostic factors in these patients.

**067 Fertility in Young Adults Following Chemotherapy for High-Grade Bone Sarcomas**

Richard D Lackman, Kathy Henderson, Rakesh Donthineni-Rao
*Department of Orthopaedics University of Pennsylvania*

*Objective:* Little has been reported in the literature regarding the effects of chemotherapy on the fertility of young patients treated for high-grade bone sarcomas.

*Methods:* We retrospectively reviewed patients with osteosarcoma and Ewing's sarcoma treated by the senior author, from 1985-94. The chemotherapeutic agents included adriamycin, cisplatin, methotrexate, ifosfamide, cytoxan, and vincristine. Patients were queried about attempts at childbirth and history of pregnancy.
*Results:* Positive reports included 10 males and 5 females with a mean age at diagnosis of 18 years (range 12 to 26). The mean time to the first conception following cessation of chemotherapy was 5.5 years (range 1 to 11). All the couples except one, attempting conception were successful without any complications during the pregnancy and without any congenital abnormalities of the babies. The unsuccessful couple underwent a battery of testing and the partner without a history of cancer was reported to be infertile. None of the couples utilized fertility-enhancing drugs.
*Conclusion:* While some articles have suggested that the effects of chemotherapy on adolescents and young adults with high-grade sarcomas are transient, actual pregnancies have not been studied. We found successful conception and subsequent birth of normal children, and should be the expectation for these patients.

### 074 The Mutation Analysis of NFAT1 in Chondrosarcoma

Tomoki Aoyama[1], Satoshi Nagayama[3], Takeshi Okamoto[1], Taisuke Hosaka[1], Takeharu Nakamata[1], Koichi Nishijo[1], Tomitaka Nakayama[2], Takashi Nakamura[2], Junya Toguchida[1]
*[1]Institiute for Frontier Medical Science Kyoto University, [2]Department of Orthopaedics Kyoto University, [3]Department of Surg. Surgical Basic Science Kyoto University*

*Objective:* NFAT (nuclear factor of activated T cell) is a family of transcription factor regulating gene expression in the immune system. In NFAT1(-/-) mice, however, ectopic proliferation of cartilageous tissues were observed, some of which resembled with chondrosarcomas (Ranger, et al. 2000). These results suggest that in addition to the role in the immune system, NFAT1 is an important regulator in chondrocyte proliferation/differentiation, and may have a role as a tumor suppressor gene in chondrosarcomas. From this standpoint, we investigated the mutation analysis of the NFAT1 gene in human chondrosarcomas.
*Methods:* From the cDNA sequence and GeneBank human genome information, we were able to determine the genomic structure of NFAT1 gene, which consisted of 10 exons. Fifteen sets of PCR primers were designed to amplify the entire coding sequences including intron sequences at exon-intron boundaries. Initial screening was performed by PCR-SSCP in 26 cases of chondrosarcomas, and samples showing abnormal bands were further investigated by direct sequence. The expressions of NFAT1 in tumor samples were analyzed by RT-PCR in 15 of 26 cases. The expressions of NFAT1 was also analyzed in 10 cases of osteosarcoma, 23 cases of soft tissue sarcomas, and 4 cases of lipomas. Cultured cell lines of osteosarcomas and mesenchymal stem cell (MSC) were also analyzed.
*Results:* We found single base change in the coding sequences at five sites, of which three, C373G(Val51Val), C1015G(Pro265Pro), C1723A(Ile501Ile) did not alter the coding amino acid. In other two sites, A1557T (His446Leu), C2859T(Pro880Leu), identical base changes were present in patients normal somatic cells, denying the possibilities of somatic mutations. Therefore, all of five base changes seemed to be single nucleotide polymorphisms, and not disease-relating mutations. The expression of NFAT1 was observed in all of examined chondrosarcomas (15/15). In addition, NFAT1 were also expressed in 9/10 osteosarcomas, 5/5 synovial sarcomas, 3/5 leiomyosarcomas, 2/3 myxoid liposarcomas, 1/3 well differentiated liposarcomas, 1/3 MFH and 1/4 lipomas. We also found the expression of NFAT1 in most of osteosarcoma cell lines (6/7), indicating that messages found in primary samples were not merely derived from infiltrating lymphocytes. The expression of NFAT1 was detected in undifferentiated MSC, and retained after the induction of chondrogenesis or osteogenesis, but disappeared after the induction of adipogenesis.
*Conclusion:* The results in this study suggest that the NFAT1 gene is unlikely to be a tumor suppressor in chondrosarcoma. Because the expression was detected in various mesenchymal tumors and also MSC, the NFAT1 may play a role in proliferation and/or differentiation of mesenchymal cells. Down-regulation of the expression after adipogenic induction in MSC, and lower expression in tumors derived from adipose tissues suggest that one of the roles of NFAT1 in mesenchymal tissues is the inhibition of adipogenesis.

### 075 Novel Type of EWS-Chop Fusion Gene in Two Cases of Liposarcomas with Aggressive Clinical Features and Atypical Histopathological Findings

Tomitaka Nakayama[1], Taisuke Hosaka[2], Yasuaki Nakashima[3], Katsuyuki Kusuzaki[4], Takeharu Nakamata[2], Tomoki Aoyama[2], Takeshi Okamoto[2], Koichi Nishijo[2], Takashi Nakamura[1], Junya Toguchida[2]
*[1]Department of Orthopaedic Surgery Kyoto University, [2]UniversityInstitiute for Frontier Medical Science Kyoto University, [3]Department of Pathology Kyoto University, [4]Department of Orthopaedic Surgery Kyoto Prof.Univ.of Med.*

*Objective:* Chimeric proteins consisting of TLS and CHOP or EWS and CHOP are characteristic markers for myxoid liposarcomas (MLS). At least nine different structure of TLS-CHOP fusion transcripts due to different breakpoints or alternative splicing were reported, whereas only one type consisting of exon 1 to 7 of EWS and exon 2 to 4 of CHOP gene was so far reported in the case of EWS-CHOP fusion gene. Here we described our analyses of 21 MLS cases, and found a novel type of EWS-CHOP fusion gene in two cases with unique clinical and histopathological findings.
*Methods:* RNAs and DNAs were extracted from surgically resected tumor tissues of 21 MLSs. RNAs with appropriate quality were available in 18 cases, and cDNAs were synthesized from them. Using these cDNAs as a template, either TLS or EWS-specific primer was used in combination of the CHOP primer to amplify TLS-CHOP or EWS-CHOP fusion genes, respectively. Amplified fragments were directly sequenced to determine the structure of fusion genes. In the remaining three cases, genomic long-distance PCR was performed to amplify genomic fragments encompassing the genomic fusion points.
*Results:* By the combination of RT-PCR and genomic long-distance PCR analysis, we found either TLS-CHOP or EWS-CHOP fusion genes in all of 21 cases. Among them, 17 cases (81%) were found to have TLS-CHOP fusion genes. The structure of these fusion genes were type 1 in five cases, type 2 in eight, type 3 in one, type 4 in two and type 5 in one case according to the classification of previous reports including ours. EWS-CHOP fusion genes were detected in four cases (19%), of which two were found to consist of exon 1 to 7 of EWS and exon 2 to 4 of CHOP, This structure is identical with those of five cases with EWS-CHOP fusion genes in previous literatures. The structure of the other two cases was found to be a novel one, consisting of exon 1 to 10 of EWS and exon 2 to 4 of CHOP gene. The clinical and pathological findings of these two cases with this novel EWS-CHOP fusion gene were quite remarkable in contrast with other cases. Both presented a rapid growing huge mass, showing good response to preoperative chemotherapy, but developing local recurrence within 12 months. Tumor tissues showed monotonous proliferation of small cells with minimum adipocytic features in abundant myxoid matrix and few vascular structures. These features were not compatible with classic myxoid or round cell liposarcoma, and also clearly different from pleomorphic or well-differentiated liposarcoma.

*Conclusion:* The fact that clinical and histopathological findings of two cases with a novel EWS-CHOP gene shared several unique features suggest the unique property of this type of rare fusion gene, which may be related to develop a distinct type of liposarcomas.

**081 Pulmonary Metastasis in STS**

Matthias Peiper, Thomas Streichert, Antja Heinecke, Jacob R. Izbicki, Wolfram Trudo Knoefel
*Dep. of General Surgery, University Hospital Hamburg-Eppendorf, Germany*

*Objective:* The lungs compromise a predilection site for pulmonary STS metastasis.
*Methods:* A retrospective analysis of all patients operated on pulmonary metastasis of STS between 1988 and 1999 was performed. Patients and tumor characteristics as well as surgical and pathological results where evaluated.
*Results:* 52 patients (31 female, 21 male) with a median age of 44 (18-75) years were operated. Primary tumors included leiomyosarcomas (n=13, 23%), MFH (n=10, 19%), MPNST (n=6, 13%) and 23 tumors of 11 other entities 42% of patients have been already treated with primary STS in our institution. Primary tumors were located subcutaneous in 12 patients, subfascial in 25 patients and in 15 patients in parenchymatous organs. Half of primary STS were poorly differentiated, while 30% were moderate and 20% were well differentiated. 29% of primary tumors had been resected achieving wide margins (R0), 71% R1. In 12.5% of patients, distant metastasis were present at initial diagnosis. In 40 patients chemotherapy had been administered (not in randomized trials), 26 of these after diagnosis of pulmonary metastasis. In 28 patients local recurrence occurred, in 25 of these before or simultaneously with pulmonary metastasis. Patients were operated by thoracotomy or sternotomy no patient was operated using thoracoscopy. Up to 4 operations were performed per patient and up to 38 pulmonary tumors were resected. At the end of follow-up 14 patients are alive, while 38 died due to tumor disease. Mean over all survival time was 37 months and 20 months after metastasectomy. No significant statistic parameters were found associated with reduced survival.
*Conclusion:* Though most patients with pulmonary STS metastasis will eventually die due to tumor disease, cures are noted and over all survival is better than and most other malignancies. Even recurrent metastasectomy may be indicated in selected patients.

**083 The Immediate Re-Excision of Soft Tissue Sarcomas After Inadequate Initial Surgery**

Thomas Streichert, Matthias Peiper, Antje Heinecke, Wolfram Trudo Knoefel, Jacob R. Izbicki
*Dep. of General Surgery, University Hospital Hamburg-Eppendorf*

*Objective:* Soft tissue sarcoma (STS), a rare tumor entity, is often mistreated by local excision because of their supposed benignity. This results in local recurrence-rates up to 70%. Since 1988 we perform a re-excision in patients where initial resection was made not achieving wide margins. Most patients were referred for assumed adjuvant therapy, but interdisciplinary evaluation of patients and charts revealed initial close resection margins.
*Methods:* From 1988 until 1998 287 patients were treated in our department suffering from primary soft tissue sarcomas of the extremities or of the trunk. 115 patients were treated before with simple excision not achieving adequate margins. These patients were re-resected in-between the next 9 to 37 days after primary resection.
*Results:* 67 male and 48 female patients with a mean age of 47 years were included into this study. The STS were located either at the extremities (lower extremity: n=67, upper extremity: n=26) or at the trunk (n=22). 62 of them were subcutaneous, 53 subfascial. The histological grade was in n=45 cases G1, in n=40 G2 and in n=30 G3. All of the subcutaneous and 33 subfascial STS were treated locally by wide resection, 20 of the latter received a compartmental resection. In 102 patients a R0-resection was achieved, 13 resection R1 patients – refused further surgery, resulting in amputation. In 53 cases (46%) the histology revealed residual tumor: 14 of them multifocally and in 50 specimen tumor was detected macroscopically. Adjuvant therapy was administered in 33 patients and chemotherapy in 12 patients. Local recurrence occurred in 15 patients (13%). After a mean follow-up of 69 (17–121) months, 87 patients were alive without evidence of disease, and 7 were alive with tumors. 8 patients died tumor-related, 6 died of other reasons.
*Conclusion:* In case of inadequate initial surgery the primary re-excision should have highest priority in the multimodality treatment concept, since nearly 50% showed a residual tumor though were reported for being excised completely. The resection with wide margins plays the key role for soft tissue sarcoma therapy leading to a smaller rate of recurrence and assumingly less distant metastasis.

**093 Alveolar Soft Part Sarcoma in Adults: Analysis of Clinical Features and Treatment Outcome in 19 Cases**

Helen C. Swannie, Omar Al-Muderis, J. Meirion Thomas, Clive L. Harmer, Cyril Fisher, Ian R. Judson
*Sarcoma Unit, Royal Marsden Hospital*

*Objective:* Alveolar soft part sarcoma (ASPS) is a rare tumour, accounting for approximately 1% of malignant soft tissue tumours. Most centres see very few cases and the literature pertaining to adults is sparse. The purpose of this study was to review the experience of our institution in treating adults presenting with ASPS and to assess strategies for management of metastatic disease, including systemic therapy and surgery.
*Methods:* The case records of 19 consecutive patients presenting with alveolar soft part sarcoma aged 15 years and over between 1984 and 2000 were reviewed retrospectively. Data were evaluated with respect to patient characteristics, location of primary tumour, frequency and site of metastases, recurrence, treatment outcome and death. Survival was defined as the interval between diagnosis and death or last follow-up visit. PFS was defined as the time from diagnosis to relapse, death, disease progression, or most recent follow up visit. The last follow-up visit in the group was November 16, 2000 and the median follow up duration of surviving patients is 68.1 months (range, 21.8 – 222.6).
*Results:* The 12 male and 7 female patients had a median age of 28 years (range 15 -51). 68% (13/19) of patients presented with extremity tumours. Local recurrence was rare (1 patient) but 74% of the patients (14/19) had developed metastatic disease. 47% (9/19) had pulmonary metastatic disease at presentation with 26% (5/19) developing metastatic disease more than 3 years after diagnosis. At last follow-up, 69% (13/19) were alive, 32% (6/19) were disease free and 37% (7/19) were alive with metastatic disease. No objective responses to chemotherapy were observed in the 47% (9/19) of patients who received chemotherapy for metastatic disease but of the 9 who underwent pulmonary metastasectomy, 7 achieved complete clearance, with only 2 of these recurring in the thorax after surgery. 4 patients of the 9 (44%) remain disease free at last follow-up.
*Conclusion:* ASPS is a rare disease entity in adults, characterised by a high metastatic rate but indolent disease course. There is currently no effective systemic treatment but resection of pulmonary metastases may be curative and should be pursued aggressively even if repeated metastasectomies are required.

**104 Non-Viral Gene Transfer into Human Osteosarcoma Cells Using the Non-Liposomal Lipid Effectene – a Primer for Malignant Bone Tumor Gene Therapy Strategies**

Detlev Gottschalk[1], B. Schmitz[2]
[1]*Department of Orthopedics – Connective Tissue Research, University clinic and Medical School, RWTH Aachen, and Interdisciplinary Centre for Clinical Research on Biomaterials (IZKF "Biomat.), RWTH Aachen,* [2]*Department of Clinical Chemistry and Pathobiochemistry, RWTH Aachen*

*Objective:* To elucidate the transfection efficacy of the new non-liposomal lipid Effectene on human osteosarcoma cells used for cancer treatment protocols in-vitro and the possible role of this new transfection agent for cancer gene therapy strategies.
*Methods:* For transfection, a 4.7 kB plasmid (pEGFP-C1, Clontech) was applied containing a CMV promotor and the enhanced green fluorescent protein (EGFP) gene sequence, a red shifted variant of the wild type GFP. For transfection of human primary osteoblast like cells (hOB) and a osteogenic, osteosarcoma cell line (HOS58), using a modified standard protocol by quiagen, two different plasmid concentrations (0.4 ug, 1.0 μg) were used. Controls were treated with Effectene reagent without prior DNA application. For microscopic analysis, cells were washed in HBSS and fixed in 4% paraformaldehyde for 20 min at RT, washed again in HBSS and analyzed by standard microscopy and confocal laser scan microscopy. For measuring transfection efficacy by FACScan analysis cells were washed in HBSS, trypsinized and subsequently analyzed on FACScalibur. PI (0.1 mg/ml) was added to each tube to exclude dead cells. The amount of EGFP-expression was determined in the life gate.
*Results:* A linearity of transfection efficacy could be observed with increasing amounts of the used plasmid concentrations in hOB. Transfection with 0.4 ug plasmid DNA results in mean of about 6.5% of EGFP expressing cells. The EGFP expression varied from weak to very strong fluorescence intensities. The amount of PI-positive cells was determined as about 20%. A higher plasmid DNA concentration (1.0 μg) resulted in increased amount of EGFP expressing viable cells (transfection rate about 13%), but effected also in a negative way the total amount of EGFP expressing living cells (about 18%). The transfection rate of HOS58 was about 11% and 13% respectively but higher plasmid concentration had no cell-toxic effect as in the non-malignant transformed hOB system (living cells 79% and 82% respectively). An increase of plasmid concentration had no significant effect on the transfection rate in HOS58 but significantly decreased the amount of viable hOBs. Therefore, future gene therapy strategies for malignant bone tumors should be performed with caution, even using non-viral vectors.
*Conclusion:* Effectene used as new non-viral transfection agent, here proved to be successful on human primary osteoblasts (hOB)and a human osteosarcoma cell line (HOS58) has a high reproducibility and a low cytotoxicity compared to other systems. Moreover, Effectene works in the presence of serum without restriction allowing longer incubation time. In addition, lower concentrations of DNA are needed. These items are of advantage in cancer treatment protocols. Gene therapy for malignant bone disease is a challenge for the new decade of cancer treatment and could support neoadjuvant chemotherapy and/or could help to decrease intrinsic tumor cell resistance to chemotherapeutic agents.

**115 ET-743, A New Active Drug in Adult Soft Tissue Sarcoma: A Soft-Tissue and Bone Sarcoma Group/EORTC Phase II Trial**

Axel Le Cesne[1], Jean Yves Blay[1], Ian Judson[1], Alan Van Oosterom[1], Jaap Verweij[1], John Radford[1], Paul Lorigan[1], Elisabeth Gray[1], Jose Jimeno[2], Ole Nielsen[1]
[1]*STBSG – EORTC Data Center,* [2]*Pharma Mar*

*Objective:* The main objectives of this multicenter phase II study were to assess the activity and toxicity of ET-743 (Pharma Mar/ Spain) administered at a dose of 1500 μg/m2 as 24-hour CI every 3 weeks in patients (pts) with advanced sarcoma.
*Methods:* Pts with GIST (group (gp) B) received ET-743 as front-line chemotherapy (CT) while pts with documented progressive STS (gp A and C) as 2nd/3rd line CT. Evaluation of response in gp C was performed with the new system based on RECIST criteria.
*Results:* between 5/99 and 11/00, 471 cycles of ET-743 were administered to 132 pts (47, 28 and 57 in gp A, B and C respectively). Toxicity (T) : there was no alopecia and no severe digestive T. A reversible gde 3-4 transient elevation of transaminases was seen in about 40% of pts in all gps. Febrile neutropenia (N) and gde 3-4 N were observed in 15, 7 and 3% and 45, 48 and 58% of pts included in gps A, B and C respectively. Haematological T was not cumulative (pts receiving from 1 to 14 cycles). There were 4 toxicity-related deaths in gp A. After protocol amendment (10/99) requiring normal ALP at inclusion and ET-743 dose reduction (1200 μg/m2) in case of an intercycle-rise in bil/ALP, the incidence of serious T has been significantly decreased. No OR were observed in GIST pts. Among the 94 eligible pts in gp A and C, there were 8 PR (OR rate of 9%), 48 NC (51%) (including 4 not confirmed PR and 2 prolonged major MR) and 28 PD (30%). Two patients are ongoing and 8 pts were not evaluable. The 6 months progression free survival (6PFS) for all included pts were 31, 24 and 28% in gp A, B and C respectively. Thirty six pts received at least 6 cycles of CT. The 6PFS observed in non-GIST sarcoma compares favorably with those (6PFS: 18%) obtained with other active drugs tested in 2nd line CT in previous EORTC trials (Van Glabbeke, ASCO 01). Median overall survival was 250 and 241 days for pts included in gp A and C respectively.
*Conclusion:* ET-743 is an active compound in advanced STS. Further studies with a shorter infusion time as single agent and/or in combination are warranted.

